# Only virgin type of olive oil consumption reduces the risk of mortality. Results from a Mediterranean population-based cohort

**DOI:** 10.1038/s41430-022-01221-3

**Published:** 2022-10-14

**Authors:** Carolina Donat-Vargas, Esther Lopez-Garcia, José R. Banegas, Miguel Á. Martínez-González, Fernando Rodríguez-Artalejo, Pilar Guallar-Castillón

**Affiliations:** 1 Department of Preventive Medicine and Public Health, School of Medicine, Universidad Autónoma de Madrid-IdiPaz, CIBERESP (CIBER of Epidemiology and Public Health), 28029 Madrid, Spain; 2 ISGlobal, Barcelona, Spain; 3 Unit of Cardiovascular and Nutritional Epidemiology, Institute of Environmental Medicine, Karolinska Institutet, 171 77 Stockholm, Sweden; 4 Department of Preventive Medicine and Public Health, School of Medicine, Universidad de Navarra, 1008 Navarra, Spain; 5 CIBEROBN (CIBER of Obesity and Nutrition), 28029 Madrid, Spain; 6 Department of Nutrition, Harvard TH Chan School of Public Health, Boston, MA USA; 7 IMDEA-Food Institute, CEI UAM+CSIC, Madrid, Spain

**Keywords:** Diseases, Scientific community

## Abstract

**Background:**

Evidence on the association between virgin olive oil (OO) and mortality is limited since no attempt has previously been made to discern about main OO varieties.

**Objective:**

We examined the association between OO consumption (differentiating by common and virgin varieties) and total as well as cause-specific long-term mortality

**Methods:**

12,161 individuals, representative of the Spanish population ≥18 years old, were recruited between 2008 and 2010 and followed up through 2019. Habitual food consumption was collected at baseline with a validated computerized dietary history. The association between tertiles of OO main varieties and all-cause, cardiovascular and cancer mortality were analyzed using Cox models.

**Results:**

After a mean follow-up of 10.7 years (129,272 person-years), 143 cardiovascular deaths, and 146 cancer deaths occurred. The hazard ratio (HR) (95% confidence interval) for all-cause mortality in the highest tertile of common and virgin OO consumption were 0.96 (0.75–1.23; P-trend 0.891) and 0.66 (0.49–0.90; P-trend 0.040). The HR for all-cause mortality per a 10 g/day increase in virgin OO was 0.91 (0.83–1.00). Virgin OO consumption was also inversely associated with cardiovascular mortality, with a HR of 0.43 (0.20–0.91; P-trend 0.017), but common OO was not, with a HR of 0.88 (0.49–1.60; P-trend 0.242). No variety of OO was associated with cancer mortality.

**Conclusion:**

Daily moderate consumption of virgin OO (1 and 1/2 tablespoons) was associated with a one-third lower risk of all-cause as well as half the risk of cardiovascular mortality. These effects were not seen for common OO. These findings may be useful to reappraise dietary guidelines.

## Introduction

The Mediterranean diet (MedDiet) represents the dietary pattern that was typically consumed among populations bordering the Mediterranean Sea. This pattern has been strongly and consistently associated with healthy ageing [1] and with a reduced risk of mortality, in addition to other health outcomes, such as cardiovascular disease (CVD), type 2 diabetes and cancer [1–5]. The traditional MedDiet is characterized by a high intake of olive oil (OO), fruits, nuts, vegetables, and cereals; a moderate intake of fish and poultry; a low intake of dairy products, red meat, processed meats, and sweets; as well as a moderate consumption of wine at meal times [6].

OO is not only the main culinary and dressing fat in Mediterranean countries, but also sets the MedDiet apart from other healthy dietary patterns. There is some observational evidence that OO may play a major role in explaining the associations of the MedDiet with a lower incidence of several chronic diseases [7–9], especially CVD [4, 10–12]. Virgin OO (the highest quality variety, obtained by mechanical processes and rich in phenolic compounds), has shown to have anti-inflammatory, antioxidant, and anti-atherosclerotic properties as well as beneficial effects on endothelial function and blood pressure control [8, 13–16].

In the 5-year PREDIMED clinical trial [4], which randomized 7447 older adults, cardiovascular and total mortality were respectively 38% and 10% lower among those assigned to a MedDiet supplemented with virgin OO (the goal was to consume 50 g [approximately four tablespoons] or more per day) when compared to those assigned to a reduced-fat diet. In a subsequent observational analysis of the PREDIMED population, total OO consumption at baseline was associated with reduced total and cardiovascular mortality, but no significant association was found with cancer mortality [11]. Likewise, in the preceding EPIC-Spain cohort study, both common (processed and refined) and virgin OO (unprocessed and unrefined) varieties were associated with a decreased risk of total and cardiovascular mortality but not with cancer mortality.

In recent decades, OO has become more popular outside the Mediterranean countries, even in U.S population. Thus, a recent study conducted among 60,582 women form the Nurses’ Health Study and 31,801 men from the Health Professionals Follow-up Study has found an inverse association between OO consumption and risk of total and cause-specific mortality. Compared with those who never or rarely consume total OO, those in the highest category of OO consumption (>7 g/d) had 19% lower risk of total and CVD mortality and 17% lower risk of cancer mortality [17]. In European cohorts, however, inconclusive results regarding OO consumption and mortality have been observed [18–22]. Of note is that—except for the Spanish EPIC cohort and the PREDIMED trial—none of these studies reported the results broking down by main OO varieties. This distinction is important because refined OO has much lower levels of bioactive compounds than virgin OO and may therefore have fewer health benefits.

Virgin OO contains much higher amounts of bioactive compounds like polyphenols, which have important biological properties [23–25]. Thus, as interest grows in identifying the best source of fat for human health, studies on the impact of the main OO varieties on mortality as well as the consumption amount required to generate optimal protection are warranted.

Spanish EPIC cohort was performed using convenience sampling (i.e., blood donors) and following the general design applied to all the countries in this consortium [26]; therefore, it is not representative of the Spanish population. Consequently, we aimed to assess the associations between common and virgin OO consumption and long-term risk of death (all-cause, cardiovascular, and cancer mortality) in a large and representative sample of Spanish adults.

## Methods

### Study design and population

Data were taken from the Study on Nutrition and Cardiovascular Risk in Spain (ENRICA), whose methods have been reported elsewhere [27]. In brief, 12,948 individuals ≥18 years old were selected between June 2008 and October 2010 by random stratified cluster sampling to ensure a representative sample of the non-institutionalized Spanish population. First, the sample was stratified by province and municipality size. Second, the clusters were randomly selected in two stages: municipalities and census sections. Finally, the households within each section were selected by random telephone dialing using the telephone directory as the sampling frame. Participants in the households were selected proportionally to the sex and age distribution of the Spanish population. During the first telephone call, the overall objectives and procedures of the study were explained, and selected individuals provided initial consent to participate; a formal letter of invitation and detailed written information on the study characteristics were sent to the participant’s home. Collection of blood and urine samples were included for participants’ acceptance, and the overall response rate was 51.5%. Among those not participating, the more frequent reasons were refusal to agree to a blood extraction (51.7%), no interest in the study (37.8%), and lack of time to participate (10.7%) [27].

The study was approved by the Clinical Research Ethics Committees of the La Paz University Hospital in Madrid and the Hospital Clinic in Barcelona (Spain). Written informed consent was obtained from all participants.

### Baseline data collection

At baseline (during the years 2008–2010), data on sociodemographic, lifestyle such as hours watching TV and physical activity [28], as well as morbidities were collected. Self-reported information was obtained on sex, age, educational level (no formal education, primary, and secondary or higher), and tobacco consumption (current, former, and never smoker). Weight and height were measured at home under standardized conditions, and body mass index (BMI) was calculated. The number of medications were checked against drug packages. Hypertriglyceridemia was defined as fasting plasma triglycerides ≥150 mg/dL; hypercholesterolemia as fasting plasma total cholesterol level ≥200 mg/dL or taking lipid-lowering medications; high blood pressure was defined as ≥140/90 mmHg or taking antihypertensive medication; and diabetes as self-reported or taking diabetes medication. Finally, self-reported physician-diagnosed chronic conditions (chronic obstructive respiratory disease, coronary heart disease, stroke, heart failure, osteoarthritis, cancer, and depression requiring treatment) were also collected.

### Dietary assessment

Trained and certified personnel collected information in three sequential stages: (1) a telephone interview to obtain data on sociodemographic factors, health behaviors, self-rated health, and morbidity; (2) a first home visit to collect blood samples, and (3) a second home visit to perform a physical examination, and to obtain habitual diet by using a computerized dietary history [27].

To ascertain the participant’s habitual food consumption, we used a validated computer-based dietary history (DH-ENRICA), developed from that used in the Spanish EPIC cohort [29]. It consists of a structured questionnaire administered by a trained interviewer following each eating occasion, from breakfast to just before bedtime. In the interview, respondents were asked about food consumption during the week and on the weekend, as well as seasonal variations. The DH-ENRICA collects standardized information on 880 foods and 184 recipes for dishes commonly eaten in Spain. Spanish standard food composition tables allowed for the calculation of the amount of energy and nutrients consumed [30, 31]. Study participants reported how often they had consumed different types of oils and fats, and they specified the type of oil used for cooking and dressings, as well as the oil that was part of recipes and sauces. In particular, detailed data were obtained on the consumption of common and virgin OO—comprehensively considering the dressing and cooking and frying methods.

We also calculated the Mediterranean Diet Score, based on the definition proposed by Trichopoulou et al. [32, 33] where the consumption of vegetables, legumes, fruits and nuts, cereals, and fish was considered beneficial. A value of 1 was assigned to subjects with consumption above the sex-specific median in the study sample. In contrast, consumption of red meat, processed meat and poultry, and dairy products was considered detrimental, and a value of 0 was assigned to consumption above the median. Two items, alcohol consumption (to be able to adjust the models independently for alcohol consumption without over-adjusting) and the ratio of monounsaturated/saturated fatty acids (because OO consumption is the main source of monounsaturated fatty acids (MUFAs) in the Spanish population) were not included in the index. The range of this modified index was 0 (lowest adherence) to 7 (highest).

### Mortality ascertainment

For all-cause mortality, we used the Spanish National Death Index that contains information on the vital status of all residents in Spain. Data for specific cause of death were obtained from the Statistics National Institute of Spain (https://www.ine.es/en/index.htm). All-cause deaths were obtained from baseline in 2008–2010 to the end of follow-up on January 31, 2020, while those from CVD or cancer were obtained from baseline to January 31, 2017. Follow-up was censored at the date of death or at the end of follow-up, whichever occurred first.

### Statistical analysis

Out of 13,105 participants, after excluding those that reported extreme total energy intake (800 or 5000 kcal/day for men and 500 or 4000 kcal/day for women [34]) (*n* = 884) and those with incomplete baseline dietary data (*n* = 60), a total of 12,161 participants were included in the present analysis (5708 men and 6346 women, mean age: 47 ± 17 years old).

Total OO consumption (in g per day) was estimated by adding the common and virgin varieties. OO consumption was adjusted for total energy intake by the residual’s method [35] and participants were categorized according to sex-specific tertiles of total, common, and virgin OO consumption.

To assess the associations between OO consumption and all-cause, cardiovascular and cancer mortality, Cox proportional hazard models were fitted, with attained age as the underlying timescale (birth date as origin). Hazard ratios (HRs) and their 95% confidence intervals (CI) were calculated using the lowest tertile of OO consumption as a reference or considering OO consumption as a continuous variable (per each 10 g/day, ~1 tablespoon). To investigate linear trends across tertiles of OO consumption we assigned the median value to each category and considered the variable as continuous.

We adjusted the Cox regression models for several potential confounders defined “a priori” and selected according to previous causal knowledge [36]. Thus, three models were built with progressive levels of adjustment for confounders based on information collected at enrollment. Model 1 included sex and age (continuous) and total energy intake (kcal/day). Model 2 was further adjusted for educational level, smoking status, BMI (<25, ≥25–<30, and ≥30 kg/m^2^), total physical activity (household and leisure-time activities in METs-hour/week), watching TV (hours/day), alcohol consumption (g/day), fiber intake (g/day), Mediterranean Diet Score (continuous from 0 to 7), number of medications (0, 1–3, and >3). Lastly, model 3 was also adjusted for possible mediators of the association of interest, i.e., hypertriglyceridemia (yes/no), hypercholesterolemia (yes/no), hypertension (yes/no), diabetes (yes/no), number of self-reported chronic conditions (0, 1, and ≥2). When assessing separately common and virgin OO consumption, models were further mutually adjusted. When missing values were <1% for individual covariates we used stochastic regression (which adds a random error term that appropriately reproduces the correlation between X and Y) to impute the data. All results were checked against models with complete information for all variables.

Restricted cubic spline analyses with 3 knots (at the 10th, 50th, and 90th percentiles) adjusted for the same potential confounders were represented to visually display the dose-response relationship between the consumption of total, common and virgin OO and mortality risk.

We re-ran the models for total and cardiovascular mortality including or excluding participants already diagnosed with CVD or diabetes at baseline. In addition, subgroup analyses were performed for all-cause mortality, stratifying the sample (above or below the median) by possible effect modifiers, such as age (≤ or >60 years), sex, BMI (≤ or >26.3 kg/m^2^), physical activity (≤ or >61.5 METs-h/week), as well as the adherence to the MedDiet (≤ or >score 3). P for interaction was obtained using the likelihood ratio test of the models with and without the interaction term. Finally, sensitivity analyses were performed after excluding the first 2 years of follow-up.

Analyses were carried out using STATA/SE version 16.0 (StataCorp, College Station, TX, USA). Analyses were weighted by using the svy Stata command to account for the complex sampling design. *P* values were two-tailed and *p* < 0.05 was considered as statistically significant.

## Results

A total of 12,161 participants (53% female) were followed up (129,272 person-years) during a mean of 10.7 ± 1.3 years (and 8.8 ± 1.0 for specific mortality), 739 (6.1%) all-cause, 143 (1.2%) cardiovascular, and 146 (1.2%) cancer deaths occurred. The age range of participants was 18–96.

There were no substantial differences between participants when comparing those in the lowest to the highest tertiles of total OO consumption except for age (Table 1). Those in the highest tertile were older (average 6 years of difference between extreme tertiles), more likely to consume less alcohol, more fiber, and showed a greater adherence to the MedDiet (average 1 more point in the score). A small difference was also observed in their level of education; those in the highest tertile also had a slightly higher educational level.

**Table 1:** Baseline characteristics of the cohort participants according to tertiles of total olive oil consumption in the ENRICA study = 12,161.

	Sex-specific tertiles of total olive oil consumption (g/day)^a^			
	T1 (low) (*n* = 4054)	T2 (*n* = 4054)	T3 (high) (*n* = 4053)	*P* value
Women (%)	52.6	52.6	52.6	<0.001
Age, mean (SD)	43.5 (17.0)	47.7 (16.5)	50.3 (16.1)	0.002
Total energy intake (kcal/d)	2269 (673)	2083 (622)	2227 (596)	<0.001
*Total olive oil consumption, mean (SD) (g/d)*	8.7 (4.6)	17.3 (4.6)	32.2 (10.9)	<0.001
*Common olive oil consumption, mean (SD) (g/d)*	5.5 (5.2)	11.3 (8.4)	20.2 (16.8)	<0.001
*Virgin olive oil consumption, mean (SD) (g/d)*	2.6 (4.3)	5.5 (7.8)	11.7 (15.7)	<0.001
Educational level				0.001
No formal education	28.0	28.9	30.5	
Primary	44.5	41.6	40.0	
Secondary or higher	27.5	29.5	29.6	
Smoking status (%)				<0.001
Current smoker	47.8	48.5	46.5	
Former smoker	22.4	24.8	29.0	
Never smoker	29.8	26.7	24.5	
Body mass index (%)				<0.001
<25 kg/m^2^	40.4	37.9	35.8	
≥25–<30 kg/m^2^	39.2	40.3	40.8	
≥30 kg/m^2^	20.4	21.8	23.4	
Physical activity (METs-hour/week)	67.2 (41.4)	68.4 (41.2)	68.9 (41.3)	0.167
TV (hours/day)	1.90 (1.41)	1.96 (1.42)	1.95 (1.34)	0.118
Alcohol consumption (g/day)	9.8 (17.7)	8.1 (14.6)	8.4 (14.4)	<0.001
Fiber intake (g/day)	22.0 (7.88)	23.0 (8.19)	25.3 (8.34)	<0.001
Mediterranean Diet Score (7-point score)^b^	2.9 (1.35)	3.6 (1.40)	4.0 (1.39)	<0.001
Number of medications (%)				<0.001
0	65.9	64.3	63.4	
1–3	26.8	27.6	28.3	
>3	7.3	8.1	8.3	
Hypertriglyceridemia (%)	18.0	18.1	18.5	0.864
Hypercholesterolemia (%)	47.2	52.2	55.3	<0.001
Hypertension (%)	28.9	32.9	36.9	<0.001
Prevalent diabetes (%)	11.1	11.2	12.1	<0.001
Number of chronic conditions^c^ (%)				<0.001
0	70.2	66.5	64.4	
1	22.5	23.7	25.1	
≥2	7.4	9.8	10.1	

Continuous variables presented as mean ± standard deviation and categorical variables as percentage and number of participants. *P* values were obtained using chi square test for categorical variables and ANOVA for continuous variables. Percentages may not sum to 100 because of rounding.

^a^Energy adjusted by the residual’s method.

^b^Modified score excluding intake of alcohol and ratio monounsaturated/saturated fats.

^c^Chronic obstructive respiratory disease, coronary heart disease, stroke, heart failure, osteoarthritis, cancer, or depression diagnosed by a physician.

The means (±SD) of total OO consumption (in g/day) in extreme tertiles were T1: 8.7 (±4.6) and T3: 32.2 (±10.9); for common OO consumption: T1: 0.5 (±1.4) and T3: 26.6 (±10.8); and for virgin OO consumption T1: 0.01 (±0.09); T3: 19.1 (±11.5) (Table 2).

**Table 2:** All-cause mortality according to baseline olive oil consumption in the ENRICA study = 12,161.

	Energy-adjusted tertiles of total olive oil				
	T1 (low) (*n* = 4054)	T2 (*n* = 4054)	T3 (high) (*n* = 4053)	P for trend	Per 10 g/day increase
*Mean total olive oil, g/day*	8.7 ± 4.6	17.3 ± 4.6	32.1 ± 10.9		
Deaths	222	248	269		
Person-years	46,103	43,425	39,743		
*Model 1,* HR (95% CI)	1 (Ref.)	0.90 (0.72–1.11)	0.87 (0.72–1.05)	0.178	0.93 (0.86–1.00)
*Model 2,* HR (95% CI)	1 (Ref.)	0.89 (0.71–1.10)	0.89 (0.73–1.09)	0.306	0.94 (0.88–1.01)
*Model 3,* HR (95% CI)	1 (Ref.)	0.86 (0.69–1.08)	0.88 (0.72–1.08)	0.306	0.94 (0.87–1.01)
	**Energy-adjusted tertiles of common olive oil**				
	**T1 (low) (*n*** = **4054**)	**T2 (*n*** = **4054**)	**T3 (high) (*n*** = **4053**)	**P for trend**	**Per 10** **g increase**
*Mean common olive oil, g/day*^a^	0.5 ± 1.4	9.9 ± 4.4	26.6 ± 10.8		
Deaths	216	245	278		
Person-years	43,427	45,030	40,815		
*Model 1,* HR (95% CI)	1 (Ref.)	1.12 (0.90, 1.40)	1.14 (0.94, 1.39)	0.202	1.01 (0.96, 1.07)
*Model 2,* HR (95% CI)	1 (Ref.)	0.93 (0.72, 1.22)	0.96 (0.75, 1.23)	0.897	0.97 (0.91, 1.04)
*Model 3,* HR (95% CI)	1 (Ref.)	0.94 (0.72, 1.22)	0.96 (0.75, 1.23)	0.891	0.97 (0.90, 1.04)
	**Energy-adjusted tertiles of virgin olive oil**				
	**T1 (low) (*n*** = **4054**)	**T2 (*n*** = **4054**)	**T3 (high) (*n*** = **4053**)	**P for trend**	**Per 10** **g increase**
*Mean virgin olive oil, g/day*^b^	0.01 ± 0.09	0.6 ± 1.4	19.1 ± 11.5		
Deaths	184	340	215		
Person-years	42,068	45,239	41,965		
Model 1, HR (95% CI)	1 (Ref.)	0.76 (0.58, 0.99)	0.68 (0.53, 0.88)	0.026	0.91 (0.84, 0.98)
Model 2, HR (95% CI)	1 (Ref.)	0.75 (0.57, 0.99)	0.67 (0.50, 0.90)	0.057	0.91 (0.83, 1.00)
Model 3, HR (95% CI)	1 (Ref.)	0.77 (0.58, 1.01)	0.66 (0.49, 0.89)	0.040	0.91 (0.83, 1.00)

Cox regression models were used to assess the risk of mortality by baseline energy-adjusted tertiles of olive oil (g/day) and per 10 g/day increase. Results were presented as Hazard Ratios (95% Confidence Intervals). Continuous variables presented as mean ± standard deviation.

*HR* Hazard ratio, *CI* Confidence Intervals.

*Model 1* adjusted for sex and age (continuous) and total energy intake (kcal/day).

*Model 2* further adjusted for educational level (no formal education, primary, and secondary or higher), smoking status (current, former, and never smoker), BMI (<25, ≥25–<30, and ≥30 kg/m^2^), physical activity (household and leisure-time activity in METs-hour/week), TV (hours/day), alcohol consumption (g/day), fiber intake (g/day), Mediterranean diet (seven-point score, excluding alcohol and ratio monounsaturated/saturated fats), number of medications (0, 1–3, and >3).

*Model 3* further adjusted for hypertriglyceridemia (yes/no), hypercholesterolemia (yes/no), hypertension (yes/no), diabetes (yes/no), number of self-reported chronic conditions (0,1, and ≥2).

^a^Additionally adjusted for virgin olive oil consumption (g/day).

^b^Additionally adjusted for common olive oil consumption (g/day).

### All-cause mortality

After fully adjusting for potential confounders, total OO consumption was associated with a non-significant 11% reduction in all-cause mortality (HR 0.89; 05% CI 0.72–1.08; P-for trend 0.306), when extreme tertiles were compared. There was a 6% (HR 0.94; CI 0.87–1.01) decreased risk for each 10 g/day increase in total OO consumption (one tablespoon). Furthermore, a decrease in mortality risk was observed in the highest tertile of virgin OO consumption (HR 0.66; CI 0.49–0.90; P-for trend 0.040). There was a 9% (HR 0.91; CI 0.83–1.00) lower risk of total death for each 10 g/day of virgin OO consumed. However, no association was found for common OO consumption (HR 0.96; CI 0.75–1.23; P-for trend 0.891) (Table 2 and Supplementary Fig. 1).

### Cardiovascular mortality

A 45% (HR 0.55; CI 0.35–0.85; P-for trend 0.009) and a 57% (HR 0.43; CI 0.20–0.91, P-for trend 0.017) reduction in the risk of cardiovascular death was found when comparing individuals in the extreme tertiles of total and virgin OO consumption respectively. Per each 10 g/day increase, the HR of cardiovascular mortality was 0.87; CI 0.73–1.04 for total OO, and 0.78; 0.59–1.03 for the virgin variety. Common OO was not associated with cardiovascular death (Table 3, and Supplementary Fig. 2).

**Table 3:** Cardiovascular mortality according to baseline olive oil consumption in the ENRICA study = 12,161.

	Energy-adjusted tertiles of total olive oil				
	T1 (low) (*n* = 4054)	T2 (*n* = 4054)	T3 (high) (*n* = 4053)	P for trend	Per 10 g increase
*Mean total olive oil, g/day*	8.69 ± 4.61	17.3 ± 4.61	32.1 ± 10.9		
Cardiovascular deaths	53	49	41		
Person-years	38,057	35,830	32,784		
*Model 1,* HR (95% CI)	1 (Ref.)	0.74 (0.45, 1.16)	0.54 (0.35, 0.84)	0.007	0.85 (0.71, 1.01)
*Model 2,* HR (95% CI)	1 (Ref.)	0.72 (0.46, 1.18)	0.55 (0.35, 0.85)	0.009	0.87 (0.73, 1.04)
*Model 3,* HR (95% CI)	1 (Ref.)	0.69 (0.42, 1.13)	0.55 (0.35, 0.85)	0.009	0.87 (0.73, 1.04)
	**Energy-adjusted tertiles of common olive oil**				
	**T1 (low) (*n*** = **4054**)	**T2 (*n*** = **4054**)	**T3 (high) (*n*** = **4053**)	**P for trend**	**Per 10** **g increase**
*Mean common olive oil, g/ day*^a^	0.47 ± 1.40	9.90 ± 4.45	26.6 ± 10.8		
Cardiovascular deaths	35	60	48		
Person-years	35,810	37,185	33,675		
*Model 1,* HR (95% CI)	1 (Ref.)	1.84 (1.14, 2.96)	1.20 (0.72, 2.00)	0.800	0.99 (0.87, 1.11)
*Model 2,* HR (95% CI)	1 (Ref.)	1.31 (0.77, 2.25)	0.87 (0.49, 1.56)	0.220	0.89 (0.76, 1.06)
*Model 3,* HR (95% CI)	1 (Ref.)	1.32 (0.77, 2.28)	0.88 (0.49, 1.60)	0.242	0.90 (0.76, 1.06)
	**Energy-adjusted tertiles of virgin olive oil**				
	**T1 (low) (*n*** = **4054**)	**T2 (*n*** = **4054**)	**T3 (high) (*n*** = **4053**)	**P for trend**	**Per 10** **g increase**
*Mean virgin olive oil, g/ day*^b^	0.01 ± 0.089	0.59 ± 1.40	19.1 ± 11.5		
Cardiovascular deaths	32	79	32		
Person-years	34,589	37,494	34,587		
*Model 1,* HR (95% CI)	1 (Ref.)	0.78 (0.41, 1.50	0.52 (0.27, 1.00)	0.035	0.81 (0.64, 1.02)
*Model 3,* HR (95% CI)	1 (Ref.)	0.76 (0.39, 1.50)	0.45 (0.21, 0.94)	0.023	0.78 (0.59, 1.03)
*Model 2,* HR (95% CI)	1 (Ref.)	0.76 (0.39, 1.48)	0.43 (0.20, 0.91)	0.017	0.78 (0.59, 1.03)

Cox regression models were used to assess the risk of mortality by baseline energy-adjusted tertiles of olive oil (g/day) and per 10 g/day increase. Results were presented as Hazard Ratios (95% Confidence Intervals). Continuous variables presented as mean ± standard deviation.

*HR* Hazard ratio, *CI* Confidence Intervals.

*Model 1* adjusted for sex and age (continuous) and total energy intake (kcal/day).

*Model 2* further adjusted for educational level (no formal education, primary, and secondary or higher), smoking status (current, former, and never smoker), BMI (<25, ≥25–<30, and ≥30 kg/m^2^), physical activity (household and leisure-time activity in METs-hour/week), TV (hours/day), alcohol consumption (g/day), fiber intake (g/day), Mediterranean diet (7-point score, excluding alcohol and ratio monounsaturated/saturated fats), number of medications (0, 1 to 3, and >3).

*Model 3* further adjusted for hypertriglyceridemia (yes/no), hypercholesterolemia (yes/no), hypertension (yes/no), diabetes (yes/no), number of self-reported chronic conditions (0,1, and ≥2).

^a^Additionally adjusted for virgin olive oil consumption (g/day).

^b^Additionally adjusted for common olive oil consumption (g/day).

### Cancer mortality

Neither the consumption of total nor virgin OO was associated with cancer mortality. The HR comparing extreme tertiles of total OO consumption was 1.03; CI 0.61–1.74, P-for trend 0.924 (Table 4).

**Table 4:** Cancer mortality according to baseline olive oil consumption in the ENRICA study = 12,161.

	Energy-adjusted tertiles of total olive oil				
	T1 (low) (*n* = 4054)	T2 (*n* = 4054)	T3 (high) (*n* = 4053)	P for trend	Per 10 g increase
*Mean total olive oil, g/day*	8.69 ± 4.61	17.3 ± 4.61	32.1 ± 10.9		
Cancer deaths	38	56	52		
Person-years	38,057	35,830	32,784		
*Model 1,* HR (95% CI)	1 (Ref.)	1.26 (0.81, 1.95)	1.01 (0.63, 1.65)	0.886	0.97 (0.83, 1.13)
*Model 2,* HR (95% CI)	1 (Ref.)	1.30 (0.82, 2.05)	1.04 (0.62, 1.74)	0.931	0.97 (0.84, 1.12)
*Model 3,* HR (95% CI)	1 (Ref.)	1.28 (0.80, 2.05)	1.03 (0.61, 1.74)	0.924	0.97 (0.83, 1.12)
	**Energy-adjusted tertiles of common olive oil**				
	**T1 (low) (*n*** = **4054**)	**T2 (*n*** = **4054**)	**T3 (high)** ***(n*** = **4053**)	**P for trend**	**Per 10** **g increase**
*Mean common olive oil, g/ day*^*a*^	0.47 ± 1.40	9.90 ± 4.45	26.6 ± 10.8		
Cancer deaths	50	39	57		
Person-years	35,810	37,185	33,675		
*Model 1*, HR (95% CI)	1 (Ref.)	0.78 (0.50, 1.21)	1.02 (0.66, 1.57)	0.831	1.01 (0.89, 1.14)
*Model 2*, HR (95% CI)	1 (Ref.)	0.68 (0.40, 1.15)	0.86 (0.49, 1.52)	0.995	0.98 (0.85, 1.13)
*Model 3*, HR (95% CI)	1 (Ref.)	0.67 (0.39, 1.15)	0.86 (0.49, 1.51)	0.999	0.98 (0.85, 1.13)
	**Energy-adjusted tertiles of virgin olive oil**				
	**T1 (low) (*n*** = **4054**)	**T2 (*n*** = **4054**)	**T3 (high) (*n*** = **4053**)	**P for trend**	**Per 10** **g increase**
*Mean virgin olive oil, g/ day*^b^	0.01 ± 0.089	0.59 ± 1.40	19.1 ± 11.5		
Cancer deaths	34	60	52		
Person-years	34,589	37,494	34,588		
*Model 1*, HR (95% CI)	1 (Ref.)	1.02 (0.54, 1.91)	1.15 (0.67, 1.96)	0.533	0.97 (0.82, 1.13)
*Model 2*, HR (95% CI)	1 (Ref.)	0.94 (0.44, 1.99)	1.19 (0.73, 1.92)	0.334	0.97 (0.81, 1.17)
*Model 3*, HR (95% CI)	1 (Ref.)	0.91 (0.43, 1.95)	1.19 (0.73, 1.93)	0.322	0.98 (0.81, 1.18)

Cox regression models were used to assess the risk of mortality by baseline energy-adjusted tertiles of olive oil (g/day) and per 10 g/day increase. Results were presented as Hazard Ratios (95% Confidence Intervals). Continuous variables presented as mean ± standard deviation.

*HR* Hazard ratio, *CI* Confidence Intervals.

*Model 1* adjusted for sex and age (continuous) and total energy intake (kcal/day).

*Model 2* further adjusted for educational level (no formal education, primary, and secondary or higher), smoking status (current, former, and never smoker), BMI (<25, ≥25–<30, and ≥30 kg/m^2^), physical activity (household and leisure-time activity in METs-hour/week), TV (hours/day), alcohol consumption (g/day), fiber intake (g/day), Mediterranean diet (seven-point score, excluding alcohol and ratio monounsaturated/saturated fats), number of medications (0, 1–3, and >3).

*Model 3* further adjusted for hypertriglyceridemia (yes/no), hypercholesterolemia (yes/no), hypertension (yes/no), diabetes (yes/no), number of self-reported chronic conditions (0,1, and ≥2).

^a^Additionally adjusted for virgin olive oil consumption (g/day).

^b^Additionally adjusted for common olive oil consumption (g/day).

### Subgroup analyses

The reduction in all-cause mortality associated with virgin OO consumption was greater among participants with prevalent CVD or diabetes than among those free of these conditions. However, those free of CVD or diabetes and with higher virgin OO consumption exhibited a greater reduction in cardiovascular mortality (Supplementary Tables 2 and 3).

**Fig. 1:**
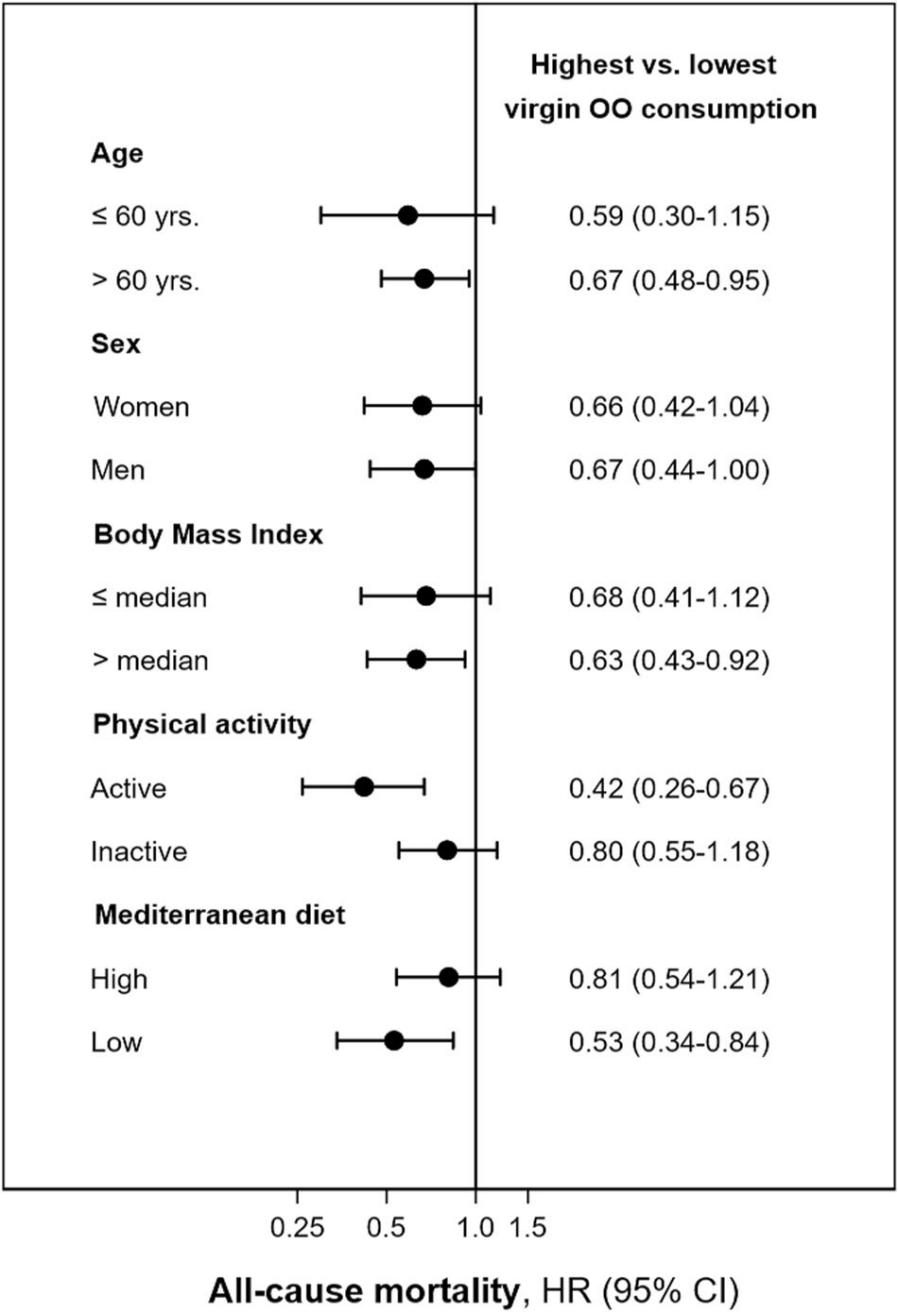
All-cause mortality risk according to tertiles of virgin olive oil consumption by body mass index, physical activity, and adherence to Mediterranean diet categories, in the ENRICA study = 12,161. Subgroup analyses were performed for total mortality, stratifying the population by possible effect modifiers such as sex, BMI (≤ or >26.3 kg/m^2^), total physical activity (≤ or >61.5 METs-hour/week) and adherence to the Mediterranean diet (≤ or >score 3). P for interaction was obtained for each subgroup analysis using the likelihood ratio test of models with and without the interaction term. Two items, alcohol and the ratio of monounsaturated/saturated fats were not included for the calculation of the Mediterranean Diet Score, thus the range in this modified score was from 0 (lowest adherence) to 7 (highest). The graph displays the hazard ratios (95% CI) for total mortality comparing the highest consumption of virgin olive oil (19 ± 12 g/day) with the lowest (<1 g/day), adjusted for sex, age (continuous), total energy intake (kcal/day), educational level (no formal education, primary, and secondary or higher), smoking status (current, former, and never smoker), BMI (<25, ≥25–<30, and ≥30 kg/m^2^), (household and leisure-time activity in METs-hour/week), TV (hours/day), alcohol consumption (g/day), fiber intake (g/day), Mediterranean diet (seven-point score, excluding alcohol and ratio monounsaturated/saturated fats), number of medications (0, 1–3, and >3), hypertriglyceridemia (yes/no), hypercholesterolemia (yes/no), hypertension (yes/no), diabetes (yes/no), number of self-reported chronic conditions (0,1, and ≥2), and common olive oil consumption (g/day). The multiplicative interaction term between virgin olive oil consumption and the subgroup variable were for age (*P* value = 0.5132), for sex (*P* value = 0.596), for BMI (*P* value = 0.333), for total physical activity (*P* value = **0.045**), and for adherence to Mediterranean Diet (*P* value = 0.129).

There was no evidence of significant interaction by sex, BMI, or adherence to the MedDiet. However, a significant multiplicative interaction between virgin OO consumption and physical activity on total mortality was identified (*P* value for interaction 0.045), with a greater risk reduction among those who were more physically active at baseline (HR 0.26; 0.26–0.67; P trend 0.007). The results also suggested that the protective effects of virgin OO against mortality could be even greater in those with lower adherence to the MedDiet. (Supplementary Table 3 and Fig. 1).

The inverse association between OO consumption and all-cause and cardiovascular mortality remained robust in sensitivity analyses after excluding the first 2 years of follow-up (data not shown).

## Discussion

In this representative sample of the Spanish adult population, while common OO was not associated with mortality, virgin OO was associated with a significant 34% reduction in all-cause and 57% cardiovascular mortality when comparing negligible consumption vs. ~20 g/day of consumption. However, OO consumption was not associated with cancer mortality. This is the first study, in which a clear benefit on all-cause and cardiovascular mortality has been observed for virgin OO but not for the common OO variety.

These findings do not completely agree with the two previous studies conducted in Mediterranean populations. In the EPIC-Spain study [26], per each 10 g/2000 kcal of total OO consumption, there was a 7% (HR 0.93; CI 0.90–0.97), a 13% (HR 0.87; CI 0.80–0.94), and a 2% (HR 0.98; CI 0.93–1.04) lower risk of all-cause, cardiovascular, and cancer mortality, respectively. Unlike our study, the risk of total mortality was similar among those exclusively consuming the common or the virgin varieties of OO. However, in this study, data was collected between 1992 and 1996 when virgin OO was consumed in lower quantities in Spain. Again, the EPIC-Spain study did not find any association between OO consumption and cancer mortality.

Similarly, in the observational analysis of the PREDIMED trial [11], for each 10 g/day increase of total OO consumption there was a 6% (HR 0.94; CI 0.87–1.00) lower risk of all-cause mortality as well as, a 13% (HR 0.87; CI 0.81–0.94), and a 5% (HR 0.95; CI 0.85–1.07) lower risk of cardiovascular and cancer mortality, respectively. The reduction in mortality risk associated with the consumption of virgin OO was slightly lower: for each 10 g/day consumed, the risks of all-cause, cardiovascular, and cancer death were 4% (HR 0.96; CI 0.91–1.01), 7% (HR 0.93; 0.84–1.03), and 3% (HR 0.97; CI 0.89–1.05) lower, respectively.

Other studies carried out in some Mediterranean countries, made no distinction among OO varieties. In the French Three-City Study that included community-based older adults (mean age: 74 years old), the benefit of OO consumption was found for total mortality only in women [19]. Moreover, no association was found between total OO consumption and all-cause mortality in a general Greek population [22]. Likewise, in a recent prospective cohort study among an Iranian population (aged ≥ 35 years old) no association was found for OO consumption nor total or cardiovascular mortality [20].

In the recent published study among >90,000 U.S. women and men [17], it was found a HR of 0.81 (95% CI: 0.78–0.84) for all-cause mortality among participants who had the highest consumption of total OO (>0.5 tablespoon/day or >7 g/day), compared with those who never or rarely consumed. Higher OO consumption was also associated with a 19% lower risk of cardiovascular mortality (HR: 0.81; 95% CI: 0.75–0.87) and 17% lower risk of cancer mortality (HR: 0.83; 95% CI: 0.78–0.89). Despite the study did not distinguish between OO varieties, it was remarkable that small amounts of total OO (<1 tablespoon/day) already reflected a significant benefit in terms of mortality. Moreover, contrary to most previous studies including ours, it was also found an inverse association between OO consumption and total cancer mortality [17].

OO is characterized by containing high amounts of MUFAs, particularly the oleic acid. Nevertheless, meta-analyses of observational studies have found that increased blood levels or intake of oleic acid or MUFAs bring no cardiovascular benefits [37, 38]. Virgin olive oil (OO), however, is also enriched by abundant bioactive compounds with a broad-spectrum of beneficial effects on health, including cardioprotective effects [39]. However, during the refining process used to obtain the common OO variety, most of these minor compounds are lost and chemical solvents are added. Therefore, these healthful compounds are basically found in sufficient amounts only in the virgin variety of OO, which is obtained only by mechanical means through crushing and pressing olives. By contrast, common OO, which is a mixture of virgin (20%) and refined (80%) OO has much fewer bioactive compounds. This may explain the clear reduction in mortality (total and cardiovascular) linked to virgin OO, but not to common OO [16, 23].

The bioactive compounds of virgin OO provide anti-inflammatory effects (decreasing C reactive protein and the expression of pro-inflammatory genes), reduce oxidative stress, regulate the blood pressure and platelet aggregation, improve lipid and glucose metabolism, bone calcification and mineralization, as well as improves the composition of the microbiota [8, 23, 24, 40]. The antitumor effects of virgin OO phenols have also been studied, including their capacity to inhibit proliferation and to promote apoptosis in several tumor cell lines, through diverse mechanisms [41].

An inverse association between the MedDiet pattern (characterized by a high intake of OO, fruit, vegetables, and whole grains) and cancer incidence and mortality has reliably reported [42]. In a recent published study among U.S population [17] reported that OO consumption was associated with overall cancer mortality, in both women and men, indicating that the association is not restricted to a potential reduction in breast cancer mortality [36]. However, our study has not found association between any variety of OO consumption and cancer mortality.

Interestingly, we reported an interaction between virgin OO consumption and physical activity. Higher virgin OO consumption (vs. lower) in combination with high total physical activity was associated with a 58% reduced risk of total mortality when comparing extreme tertiles of consumption. The possible explanation is that both—virgin OO consumption and regular physical activity—are acting together to intensely alleviate the negative effects on health caused by inflammation and oxidative stress [43].

This is the first population-based cohort study to evaluate the association between consumption of OO varieties and total and specific mortality in Spain. Other strengths are its prospective design, the adjustment for a wide array of potential confounders, the long follow-up period (approximately from 2008 to 2019), and the relatively large sample size that allowed to perform subgroup and sensitivity analyses to reinforce the findings. We distinguish between varieties of OO consumption using a validated dietary history and considering cooking, frying, and dressings, as well as OO used in recipes, dishes and sauces. Furthermore, data were collected by trained personnel during a face-to-face interview. However, some degree of misclassification may still exist, though this misclassification is likely to be non-differential, which may bias the observed associations to the null. We cannot rule out that the differences observed between common and virgin OO consumption might partly represent residual confounding due to socioeconomic status. However, Spain is the largest OO producer in the world and OO consumption is ingrained in Spanish culture encompassing all socioeconomic status. This could be a difference when performing studies on OO consumption in non-Mediterranean countries.

## Conclusion

In conclusion, we evidenced a noteworthy reduction in total and CVD mortality risk linked to high consumption of virgin OO, but not common (refined) OO. Our results also suggest a synergism between high virgin OO consumption and total physical activity on all-cause mortality risk reduction. Presently, several dietary guidelines recommend healthy plant oils (e.g., olive or canola oil) as part of the usual diet. Based on our findings, these dietary guidelines should emphasize the preferable consumption of virgin OO over the refined one. Whether virgin OO is also superior to other vegetable oils needs to be tested in future studies.

## Supplementary information

Supplement Material

## Data Availability

Data described in the paper, code book, and analytic code will be made available upon request pending application and approval.

## Code availability

The STATA code used to generate result is available on request.
