# Correction: Only virgin type of olive oil consumption reduces the risk of mortality. Results from a Mediterranean population-based cohort

**DOI:** 10.1038/s41430-022-01239-7

**Published:** 2022-11-23

**Authors:** Carolina Donat-Vargas, Esther Lopez-Garcia, José R. Banegas, Miguel Á. Martínez-González, Fernando Rodríguez-Artalejo, Pilar Guallar-Castillón

**Affiliations:** 1 Department of Preventive Medicine and Public Health, School of Medicine, Universidad Autónoma de Madrid-IdiPaz, CIBERESP (CIBER of Epidemiology and Public Health), 28029 Madrid, Spain; 2 ISGlobal, Barcelona, Spain; 3 Unit of Cardiovascular and Nutritional Epidemiology, Institute of Environmental Medicine, Karolinska Institutet, 171 77 Stockholm, Sweden; 4 Department of Preventive Medicine and Public Health, School of Medicine, Universidad de Navarra, 1008 Navarra, Spain; 5 CIBEROBN (CIBER of Obesity and Nutrition), 28029 Madrid, Spain; 6 Department of Nutrition, Harvard TH Chan School of Public Health, Boston, MA USA; 7 IMDEA-Food Institute, CEI UAM+CSIC, Madrid, Spain

**Keywords:** Diseases, Scientific community

Correction to: *European Journal of Clinical Nutrition* 10.1038/s41430-022-01221-3, published online 14 October 2022

In the conclusion of this article, the sentence “Our results also suggest a negative synergism between high virgin OO consumption and total physical activity on all-cause mortality” was revised to read “Our results also suggest a synergism between high virgin OO consumption and total physical activity on all-cause mortality risk reduction”. The original article has been corrected.

