# Supplementary material for: Only virgin type of olive oil consumption reduces the risk of mortality. Results from a Mediterranean population-based cohort
Source: Eur J Clin Nutr. 2022 Oct 14;77(2):226–34. doi: 10.1038/s41430-022-01221-3 (PMC9908537; doi:10.1038/s41430-022-01221-3)
Supplement: Supplementary file 1 — Supplement Material [file 41430_2022_1221_MOESM1_ESM.pdf]

SUPPLEMENTAL MATERIAL

Only virgin type of olive oil consumption reduces the risk of mortality. Results from a Mediterranean population-based cohort.

Carolina Donat-Vargas, Esther Lopez-Garcia, José R Banegas, Miguel Á. Martínez-González, Fernando Rodríguez-Artalejo, Pilar Guallar-Castillón

Supplemental Table 1. All-cause and cardiovascular mortality risk according to baseline virgin olive oil consumption in those subjects with prevalent cardiovascular disease or diabetes mellitus (N=998)

	**Energy-adjusted tertiles of virgin olive oil**				
	**T1 (low)** (**n**=260)	**T2** (**n**=404)	**T3 (high)** (**n**=324)		
Mean virgin olive oil, g/ day	<0.01±0.05	0.45±1.30	18.6±11.5		
**All-cause mortality**				**P for trend**	**Per 10 g increase**
Total deaths	68	106	63		
Person-years	2,467	4,160	3,336		
**Model 1**, HR (95% CI)	1 (Ref.)	0.63 (0.38, 1.06)	0.55 (0.33, 1.06)	0.066	0.90 (0.77, 1.05)
**Model 3**, HR (95% CI)	1 (Ref.)	0.56 (0.32, 0.98)	0.44 (0.25, 0.78)	0.026	0.87 (0.72, 1.06)
**Cardiovascular mortality**				**P for trend**	**Per 10 g increase**
Cardiovascular deaths	16	35	16		
Person-years	2,078	3,518	2,798		
**Model 1**, HR (95% CI)	1 (Ref.)	0.49 (0.18, 1.32)	0.43 (0.16, 1.14)	0.235	0.81 (0.58, 1.14)
**Model 3**, HR (95% CI)	1 (Ref.)	0.49 (0.16, 1.49)	0.43 (0.15, 1.26)	0.338	0.85 (0.57, 1.27)

Cox regression models were used to assess the risk of mortality by baseline energy-adjusted tertiles of olive oil (g/day) and as a continuous variable (10 g increase/day). Results were presented as Hazard Ratios (95% Confidence Intervals). Continuous variables presented as mean ± standard deviation.

Abbreviations: HR: Hazard ratio; **CI:** Confidence Intervals.

Model 1 adjusted for sex and age (continuous) and total energy intake (kcal/day)

Model 3 further adjusted for educational level (no formal education, primary, and secondary or higher), smoking status (current, former, and never smoker), BMI (<25, ≥25-<30, and ≥30 kg/m^2), physical activity in household activities and leisure time (METs-hour/week), TV (hours/day), alcohol consumption (g of ethanol/day), fiber intake (g/day), Mediterranean diet (7-point score, excluding alcohol and ratio monosaturated/saturated fats), number of medications (0, 1 to 3, and >3), hypertriglyceridemia (yes/no), hypercholesterolemia (yes/no), hypertension (yes/no), diabetes (yes/no), number of self-reported chronic conditions (0,1, and ≥2), and common olive oil consumption (g/day)

Supplemental Table 2. All-cause and cardiovascular mortality risk according to baseline virgin olive oil consumption excluding prevalent cardiovascular disease and diabetes mellitus (N=11,173)

	**Energy-adjusted tertiles of virgin olive oil**				
	**T1 (low) (n**=3,794)	**T2 (n**=3,650)	**T3 (high) (n**=3,729)		
Mean virgin olive oil, g/ day	0.01±0.09	0.60±1.41	19.1±11.5		
**All-cause mortality**				**P for trend**	**Per 10 g increase**
Total deaths	116	234	152		
Person-years	39,601	41,080	38,629		
**Model 1**, HR (95% CI)	1 (Ref.)	0.81 (0.59, 1.11)	0.75 (0.55, 1.01)	0.168	0.91 (0.83, 1.00)
**Model 3**, HR (95% CI)	1 (Ref.)	0.84 (0.61, 1.16)	0.78 (0.55, 1.13)	0.343	0.91 (0.82, 1.02)
**Cardiovascular mortality**				**P for trend**	**Per 10 g increase**
Cardiovascular deaths	16	44	16		
Person-years	32,510	33,976	31,790		
**Model 1**, HR (95% CI)	1 (Ref.)	1.14 (0.50, 2.57)	0.54 (0.23, 1.29)	0.036	0.79 (0.57, 1.09)
**Model 3**, HR (95% CI)	1 (Ref.)	1.05 (0.46, 2.39)	0.31 (0.11, 0.93)	0.006	0.66 (0.42, 1.02)

*****Excluding prevalent CVD (n=269) and diabetes mellitus (n=790)

Cox regression models were used to assess the risk of mortality by baseline energy-adjusted tertiles of olive oil (g/day) and as a continuous variable (10 g increase/day). Results were presented as Hazard Ratios (95% Confidence Intervals). Continuous variables presented as mean ± standard deviation.

Abbreviations: HR: Hazard ratio; **CI:** Confidence Intervals.

Model 1 adjusted for sex and age (continuous) and total energy intake (kcal/day)

Model 3 further adjusted for educational level (no formal education, primary, and secondary or higher), smoking status (current, former, and never smoker), BMI (<25, ≥25-<30, and ≥30 kg/m^2), physical activity in household activities and leisure time (METs-hour/week), TV (hours/day), alcohol consumption (g of ethanol/day), fiber intake (g/day), Mediterranean diet (7-point score, excluding alcohol and ratio monosaturated/saturated fats), number of medications (0, 1 to 3, and >3), hypertriglyceridemia (yes/no), hypercholesterolemia (yes/no), hypertension (yes/no), diabetes (yes/no), number of self-reported chronic conditions (0,1, and ≥2), and common olive oil consumption (g/day).

Supplemental Table 3. All-cause mortality according to tertiles of virgin olive oil consumption by age, sex, body mass index, physical activity, and adherence to Mediterranean diet in the ENRICA study=12,161

	**Energy-adjusted tertiles of virgin olive oil consumption**			
**Age**	**T1 (low)**	**T2**	**T3 (high)**	**P for trend**
**≤ 60 years**				
N	3,354	2,751	2,956	
Total deaths	48	42	38	
**Model 3**, HR (95% CI)	1 (Ref.)	0.80 (0.44, 1.46)	0.59 (0.30, 1.15)	0.174
**> 60 years**				
N	700	1,303	1,097	
Total deaths	136	298	177	
**Model 3**, HR (95% CI)	1 (Ref.)	0.76 (0.55, 1.05)	0.67 (0.48, 0.95)	0.101
**Sex**	**T1 (low)**	**T2**	**T3 (high)**	**P for trend**
**Women**				
N	2,134	2,134	2,133	
Total deaths	87	147	84	
**Model 3**, HR (95% CI)	1 (Ref.)	0.64 (0.42, 0.96)	0.66 (0.42, 1.04)	0.446
**Men**				
N	1,920	1,920	1,920	
Total deaths	97	193	131	
**Model 3**, HR (95% CI)	1 (Ref.)	0.89 (0.61, 1.30)	0.67 (0.44, 1.00)	0.029
**BMI**	**T1 (low)**	**T2**	**T3 (high)**	**P for trend**
**≤ median (26.3 kg/m^2)**				
n	2,038	1,970	1,981	
Total deaths	51	127	81	
**Model 3**, HR (95% CI)	1 (Ref.)	0.89 (0.57, 1.40)	0.68 (0.41, 1.12)	0.092
**> median (26.3 kg/m^2)**				
n	2,016	2,084	2,072	
Total deaths	133	213	134	
**Model 3**, HR (95% CI)	1 (Ref.)	0.70 (0.50, 0.98)	0.63 (0.43, 0.92)	0.119
**Total physical activity**	**T1 (low)**	**T2**	**T3 (high)**	**P for trend**
**Active**<br>> median, 61.5 METs-h/wk				
n	2,039	1,971	2,070	
Total deaths	76	92	72	
**Model 3**, HR (95% CI)	1 (Ref.)	0.55 (0.35, 0.86)	0.42 (0.26, 0.67)	0.007
**Inactive**<br>≤ median, 61.5 METs-h/wk				
n	1,908	1,835	1,840	
Total deaths	108	248	143	
**Model 3**, HR (95% CI)	1 (Ref.)	0.96 (0.68, 1.35)	0.80 (0.55, 1.18)	0.192
**Adherence to Mediterranean Diet**	**T1 (low)**	**T2**	**T3 (high)**	**P for trend**
**Score > 3**				
n	1,629	2,094	2,335	
Total deaths	86	202	136	
**Model 3**, HR (95% CI)	1 (Ref.)	0.97 (0.65, 1.43)	0.81 (0.54, 1.21)	0.176
**Score ≤ 3**				
n	2,425	1,960	1,718	
Total deaths	98	138	79	
**Model 3**, HR (95% CI)	1 (Ref.)	0.55 (0.38, 0.81)	0.53 (0.34, 0.84)	0.161

Subgroup analyses were performed for total mortality, stratifying the population by possible effect modifiers such as sex, BMI (≤ or > 26.3 kg/m^2), total physical activity (≤ or > 61.5 METs-h/week) and adherence to Mediterranean diet (≤ or > score 3). P for interaction was obtained for each subgroup analysis from the likelihood ratio test of models with and without the interaction. The items alcohol and ratio monosaturated/saturated fats were not included for the calculation of the Mediterranean Diet Score, thus the range in this modified score was 0 (lowest adherence) to 7 (highest).

Model 3 adjusted for sex, age (continuous), total energy intake (kcal/day), educational level (no formal education, primary, and secondary or higher), smoking status (current, former, and never smoker), BMI (<25, ≥25-<30, and ≥30 kg/m^2), physical activity in household activities and leisure time (METs-hour/week), TV (hours/day), alcohol consumption (g of ethanol/day), fiber intake (g/day), Mediterranean diet (7-point score, excluding alcohol and ratio monosaturated/saturated fats), number of medications (0, 1 to 3, and >3), hypertriglyceridemia (yes/no), hypercholesterolemia (yes/no), hypertension (yes/no), diabetes (yes/no), number of self-reported chronic conditions (0,1, and ≥2), and common olive oil consumption (g/day).

The multiplicative interaction term between virgin olive oil consumption and the subgroup variable were age (P value=0.5132), sex (P value=0.596), BMI (P value=0.333), total physical activity (P value=**0.045)**, and adherence to Mediterranean Diet (P value= 0.129)

Supplemental Figure 1. Splines of all-cause mortality risk according to baseline consumption (g/day) of olive oil varieties in the ENRICA study=12,161

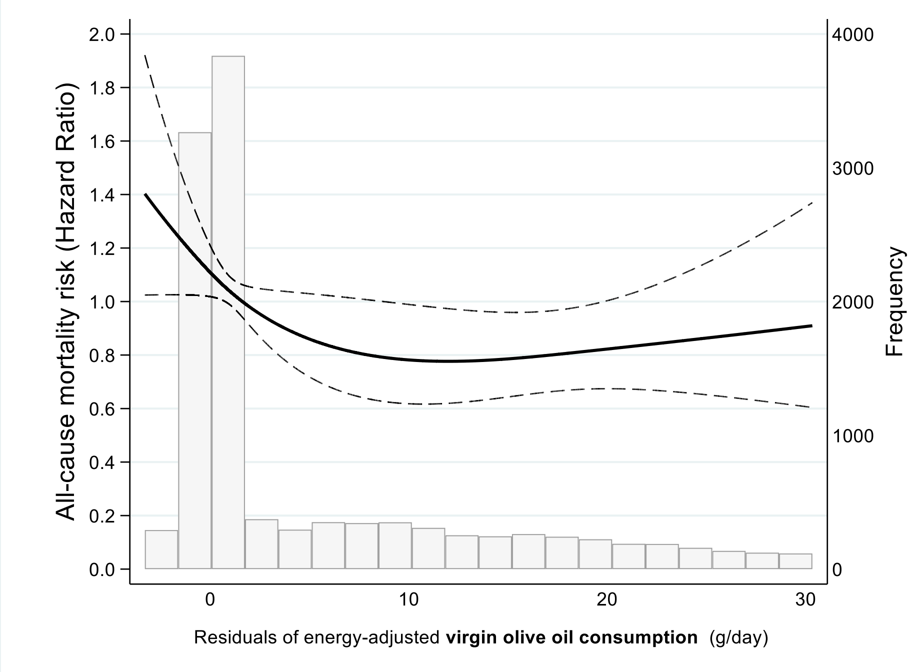

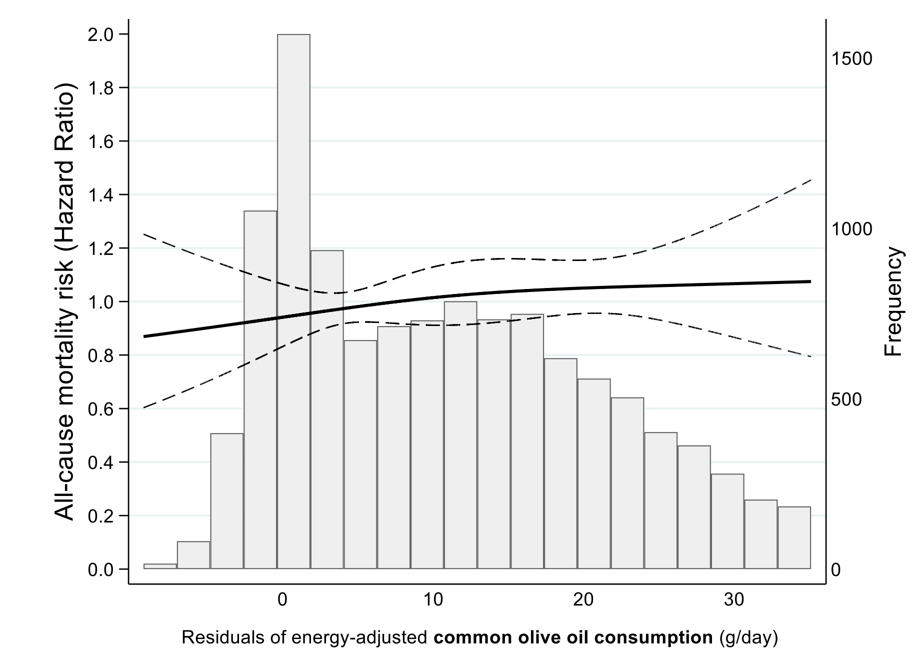

Lines are restricted cubic splines, showing the shape of the association between all-cause mortality and the consumption of olive oil varieties as continuous (g/day). The solid line represents the adjusted hazards ratios and the dashed lines indicate the lower and upper 95% CIs. The knots were located at the 10th, 50th, and 90th percentiles. Cox regression was adjusted as in model 3. Participants with an olive oil consumption above the 95th percentile were excluded for this representation.

Supplemental Figure 2. Splines of cardiovascular mortality risk according to baseline consumption (g/day) of olive oil varieties in the ENRICA study=12,161

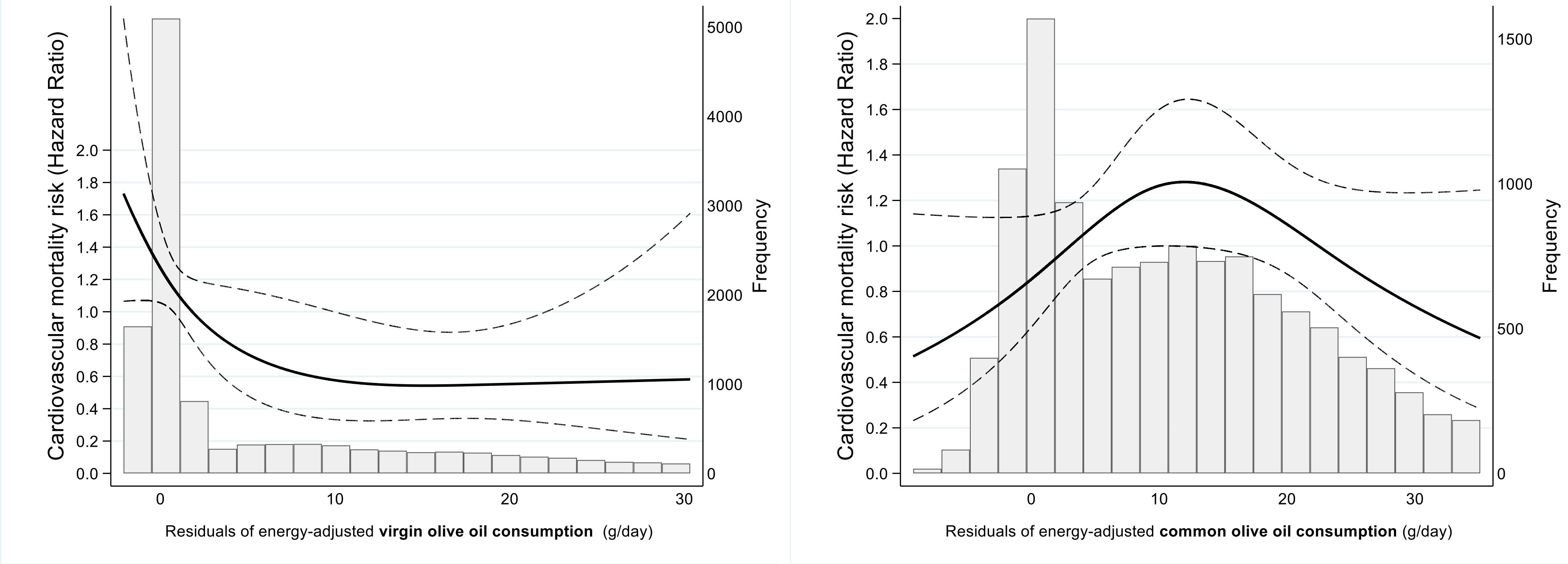

Lines are restricted cubic splines, showing the shape of the association between all-cause mortality and the consumption of olive oil varieties as continuous (g/day). The solid line represents the adjusted hazards ratios and the dashed lines indicate the lower and upper 95% CIs. The knots were located at the 10th, 50th, and 90th percentiles. Cox regression was adjusted as in model 2. Participants with an olive oil consumption above the 95th percentile were excluded for this representation.
